# MRI-based Quantification of Ossification Centers in the Human Fetal Atlas and Axis Vertebrae

**DOI:** 10.2174/0115734056429704251125042910

**Published:** 2026-03-16

**Authors:** Qinyi Han, Peng Zhao, Peng Su, Hui Zhao, Nan Lin, Mimi Tian, Lianjie Cheng, Xiangtao Lin

**Affiliations:** 1 Department of Hand & Foot and Reconstructive Microsurgery, Shandong Provincial Hospital, Shandong University, Jinan, China; 2 Department of Radiology, Shandong Provincial Hospital Affiliated to Shandong First Medical University, Jinan, China; 3 Department of Hand & Foot and Reconstructive Microsurgery, Shandong Provincial Hospital Affiliated to Shandong First Medical University, Jinan, Shandong, China; 4 Department of Radiology, Shandong Provincial ENT Hospital, Shandong University, Jinan, China; 5 Department of Medical Imaging, Shandong Public Health Clinical Center, Jinan, Shandong Province, 250021, China; 6 Department of Radiology, Shandong Provincial Hospital, Shandong University, Jinan, China

**Keywords:** Magnetic resonance imaging, Fetal cervical spine, Ossification centers, Prenatal skeletal development, Quantitative morphometry, Congenital spinal abnormalities

## Abstract

**Background:**

Although fetal MRI has been increasingly widely used in clinical and research settings, quantitative studies specifically targeting the ossification centers of the atlas and axis remain scarce. This study aims to quantitatively assess the ossification centers of the atlas and axis in fetuses using Magnetic Resonance Imaging (MRI) and establish standardized reference data for prenatal evaluation.

**Materials and Methods:**

This study included 41 human fetuses (24 males and 17 females) at 17 to 42 weeks of gestation, collected after spontaneous abortion or preterm birth that met ethical standards. High-resolution imaging was obtained by an MRI scanner. Three-dimensional volumetric data of the ossification centers were obtained and analyzed using the 3D Slicer software. Morphometric parameters, which included the 3D maximum diameter, projection surface area, and volume, were measured. Statistical analysis was conducted with SPSS 23, and growth dynamics were evaluated by regression models.

**Results:**

Analysis shows that the ossification centers of the atlas and axis increase proportionally with gestational age, and there is a significant correlation between age and measurement parameters. The average 3D maximum diameter, projected surface area, and volume show consistent growth patterns, with no significant differences between genders. The linear regression model is the best at describing developmental dynamics, with high coefficients of determination for all parameters (R ^2^>0.70).

**Discussion:**

This study indicates that the ossification centers of the fetal atlas and axis increase proportionally with gestational age, and a high correlation is observed in all morphological measurement parameters. Compared with ultrasound or CT, MRI has been proven to be a superior non-invasive imaging method that can provide high-resolution three-dimensional data for detailed evaluation of the fetal cervical spine without radiation exposure. The lack of gender-based differences supports the use of a unified growth model. These normative data provide valuable benchmarks for detecting cervical dysplasia. Although the sample size and cross-sectional design are relatively limited, this study provides clinically applicable growth references that may aid in the early diagnosis of congenital spinal abnormalities.

**Conclusion:**

This study provides standardized morphometric data for the main ossification centers of the atlas and axis in human fetuses. These findings contribute to a better understanding of fetal cervical spine development and establish a reference framework for early detection of congenital abnormalities. In addition, the research findings emphasize that MRI is a reliable and non-invasive tool for the detailed assessment of fetal skeletal maturity.

## 1 INTRODUCTION

The atlas (C1) and axis (C2) form the structural basis for head support and cervical spine movement, playing a crucial role in human posture, balance, and neuroprotection. The correct development of these vertebrae during fetal development is crucial for the normal anatomy and functional organization of the cervical spine. Abnormal development may lead to serious congenital abnormalities, such as atlantoaxial dislocation, congenital cervical deformity, and related nerve damage [1-3].

Cervical ossification begins in early fetal development, with the main ossification centers of the atlas and axis appearing in early pregnancy [4]. However, the precise time, pattern, and volume growth kinetics of these ossification centers have not been fully characterized, especially using non-invasive imaging techniques. Traditional methods such as histological examination and radiography provide valuable insights, but they are limited by sample preparation requirements, reduced soft tissue contrast, and potential artifacts [5]. In contrast, Magnetic Resonance Imaging (MRI) provides excellent soft tissue resolution, 3D visualization capabilities, and non-destructive evaluation, making it an ideal way to study fetal skeletal structure in situ [6, 7].

Although the application of fetal MRI in clinical and research settings continues to expand, there are still few quantitative studies specifically targeting the ossification centers of the atlas and axis. The existing literature mainly involves the overall development of fetal bones or a broader cervical morphology, and often lacks detailed and systematic volume analysis of these two key vertebrae [8-10]. Establishing standardized morphometric data for the ossification centers of the atlas and axis is crucial for improving prenatal diagnostic capabilities, providing information for clinical management of cervical deformities, and promoting understanding of normal fetal bone growth trajectories.

Therefore, this study aims to quantitatively evaluate the main ossification centers of the atlas and axis in human fetuses using high-resolution 3D MRI, to simulate their growth dynamics, and to generate standardized reference data across a wide range of gestational ages. By utilizing advanced MRI technology and volumetric analysis, we attempt to provide a comprehensive morphometric framework for fetal cervical spine development, offering new perspectives for research and clinical prenatal evaluation.

## 2 MATERIALS AND METHODS

The research materials included 41 human fetuses (24 males and 17 females) with gestational ages ranging from 17 to 42 weeks. These specimens were from spontaneous abortion and premature birth, and were preserved according to ethical standards before 2020. All fetuses were part of the permanent collection of normal anatomy. The research plan was approved by the Bioethics Committee of Shandong University, ensuring full compliance with ethical guidelines for research involving human fetal materials.

### 2.1 Inclusion Criteria

The inclusion criteria for this study were based on the assessment of fetal morphological integrity and the availability of recorded pregnancy history. Only when no internal or external morphological abnormalities were detected by visual inspection and fetal development was confirmed to be normal, were they included in the study. In addition, no musculoskeletal developmental defects were detected in all specimens. Gestational age was determined by measuring Crown-Rump Length (CRL) and confirmed with the maternal last menstrual period (LMP). By demonstrating a strong correlation between CRL-based and LMP-based gestational age, growth retardation was excluded (R=0.97, p<0.001). Table **1** summarizes the characteristics of the study population, including the number, gender, and gestational age distribution of fetuses.

### 2.2 Imaging Methodology

All fetal specimens were imaged using the Siemens Magnetom Prisma 3T MRI scanner (Siemens Healthcare, Erlangen, Germany) located at the Shandong Provincial Hospital. Imaging was performed to capture high-resolution, three-dimensional (3D) volumetric data for analyzing the ossification centers in the lateral and basilar regions of the occipital bone. The imaging protocol utilized a T1-weighted fast field echo sequence with the following parameters: Repetition Time (TR): 15 ms; Echo Time (TE): 7 ms; Flip Angle: 25°; Field of View (FOV): 200 mm; Slice Thickness: 0.4 mm; Matrix: 256 × 256 pixels.

Advanced imaging software (3D Slicer5.6.2) was used to process the collected MRI data in DICOM format. The scan was reconstructed in the sagittal, transverse, and coronal planes to ensure accurate identification of the ossification center. Volumetric measurements were taken on the atlas and axis.

### 2.3 Measurement Protocol

The 3D Slicer software (version 5.6.2) is used to manually segment the ossification centers of the atlas and axis layer by layer, thereby achieving three-dimensional (3D) reconstruction. Fig. (**1**) shows a representative 3D reconstruction of the ossification centers of the atlas and axis in a female fetal MRI scan at 25 weeks of pregnancy. Morphometric parameters were extracted *via* radiomics data export from 3D Slicer, including: (1) volume, (2) projection surface area, and (3) 3D maximum diameter of the ossification centers. Fig. (**2**) shows the measurement method used in this study.

Special evaluations were conducted on the following parameters for the lateral part of the atlas, the axial arch, and the axial vertebrae:

-3D maximum diameter: The maximum linear distance between two points within the 3D reconstructed image of the ossification center, accounting for the position and signal intensity of the ossified tissue.

-Projection Surface Area: The two-dimensional surface area of the projected 3D reconstruction of each ossification center, measured with adjustments for position and signal intensity.

-Volume: The total volume of each ossification center, calculated based on the signal intensity and spatial coordinates of the ossified tissue within the 3D reconstruction.

### 2.4 Statistical Analysis

All statistical analyses were conducted using SPSS Statistics software (version 23.0; IBM Corp., Armonk, NY, USA). The distribution of variables is evaluated for normality using the Shapiro-Wilk test, and the homogeneity of variance is tested using Fisher's exact test. To compare the mean values between two independent groups (*e.g*., male and female fetuses), Student's t-test was applied. For the comparison between multiple groups, one-way analysis of variance (ANOVA) was conducted, followed by Tukey's pairwise comparison post hoc test. When violating the assumptions of normality or homogeneity of variance, the non-parametric Kruskal-Wallis test was used.

Linear and curve regression analyses were conducted to evaluate the development trends of measurement parameters. The coefficient of determination (R ^2^) was used to evaluate the goodness of fit between the regression model and the observed data. We further analyzed the relationship between continuous variables using the Pearson correlation coefficient. A p-value less than 0.05 is considered statistically significant.

In order to minimize measurement and observer bias, all morphological measurements were independently conducted by two experienced researchers specializing in fetal MRI interpretation. Each parameter was measured three times at different time points under the same conditions, and the mean value was used for analysis. Intra-class correlation coefficients (ICCs) were calculated to assess Intra-observer and inter-observer reliability. These coefficients exhibit excellent consistency (*p* < 0.01), as summarized in Table **2**. These high ICC values highlight the reliability and consistency of the measurement methods used in this study.

## 3 RESULTS

No significant gender differences were observed in any measured parameters (p=0.165), therefore a unified growth model can be used for both sexes. This helps to calculate a single growth curve for each analysis parameter. The developmental dynamics of 3D maximum diameter, projected surface area, and volume show a consistent proportional increase with gestational age. These findings indicate that the ossification patterns of different genders are consistent, supporting the effectiveness of comprehensive growth analysis. Table **1** summarizes the distribution of fetal age, number, and gender.

The mean values and standard deviations for the morphometric parameters of the primary ossification centers of the lateral parts of the atlas vertebra are presented in Tables **3** and **4**. The mean 3D maximum diameter of the ossification centers ranged from 4.60 ± 0.01 mm to 20.75 ± 1.76 mm on the right side, and from 4.87 ± 0.20 mm to 20.12 ± 1.87 mm on the left, demonstrating a strong positive correlation with gestational age (*p* < 0.001, 95% CI: -4.32 to -2.78) according to the regression model: y = 0.4927 × age – 3.5465 (R^2^ = 0.9387). Similarly, the projection surface area increased from 23.24 ± 1.85 mm^2^ to 340.04 ± 45.76 mm^2^ on the right, and from 26.37 ± 3.82 mm^2^ to 333.84 ± 53.78 mm^2^ on the left, following the regression model: y = 11.192 × age – 185.39 (R^2^ = 0.8621, *p* < 0.001, 95% CI: -212.78 to -157.99). The volume measurements ranged from 6.37 ± 1.04 mm^3^ to 240.66 ± 42.76 mm^3^ on the right, and from 7.50 ± 2.13 mm^3^ to 216.98 ± 39.43 mm^3^ on the left, also showing significant linear growth with gestational age (*p* < 0.001, 95% CI: -171.97 to -119.78), modeled by: y = 7.6809 × age – 145.87 (R^2^ = 0.7645). The regression lines for these parameters are shown in Fig. (**3**).

The mean values and standard deviations for the morphometric parameters of the primary ossification centers of the axis vertebral body are presented in Table **5**. Regarding the axis vertebral body, the mean 3D maximum diameter ranged from 1.97 ± 0.14 mm to 8.41 ± 0.78 mm, fitting the regression model: y = 0.2417 × age – 1.8909 (R^2^ = 0.7775, p = 0.002, 95% CI: -3.04 to -0.74). The projection surface area increased from 7.16 ± 1.30 mm^2^ to 157.21 ± 10.75 mm^2^, modeled by: y = 5.6709 × age – 102.44 (R^2^ = 0.8478, *p* < 0.001, 95% CI: -123.86 to -81.01). The volume of the ossification center ranged from 1.46 ± 0.57 mm^3^ to 113.31 ± 8.76 mm^3^, following the model: y = 4.732 × age – 96.295 (R^2^ = 0.7095, *p* < 0.001, 95% CI: -123.29 to -69.30). The corresponding regression lines are illustrated in Fig. (**4**).

The mean values and standard deviations for the morphometric parameters of the primary ossification centers of the axis arch are presented in Tables **6** and **7**. For the lateral parts of the axis arch, the mean 3D maximum diameter ranged from 4.84 ± 0.63 mm to 20.79 ± 4.43 mm on the right, and from 5.16 ± 0.43 mm to 20.27 ± 4.06 mm on the left, demonstrating significant linear growth with gestational age (*p* < 0.001, 95% CI: -5.92 to -3.92), modeled by: y = 0.6031 × age – 4.9174 (R^2^ = 0.9316). The projection surface area ranged from 22.57 ± 6.39 mm^2^ to 435.14 ± 62.37 mm^2^ on the right, and from 25.00 ± 7.41 mm^2^ to 400.66 ± 49.87 mm^2^ on the left, described by the regression model: y = 13.549 × age – 223.56 (R^2^ = 0.8399, *p* < 0.001, 95% CI: -259.76 to -187.36). The volume measurements ranged from 5.32 ± 2.54 mm^3^ to 341.80 ± 50.54 mm^3^ on the right, and from 6.17 ± 3.43 mm^3^ to 320.24 ± 32.67 mm^3^ on the left, modeled by: y = 10.404 × age – 198.24 (R^2^ = 0.7415, *p* < 0.001, 95% CI: -235.84 to -160.64). The regression lines for the lateral parts of the axis arch are presented in Fig. (**5**).

A sensitivity analysis excluding fetuses >35 weeks was performed. The regression models remained robust, with minimal changes in slope and R^2^ values.

## 4 DISCUSSION

This study quantitatively evaluated the developmental dynamics of the ossification centers of the atlas and axis in human fetuses using high-resolution 3D MRI. The results indicate that the 3D maximum diameter, projected surface area, and volume of the ossification center exhibit a consistent proportional growth pattern relative to gestational age. No significant gender related differences were observed; a unified growth model can be used between male and female fetuses. These findings provide standardized morphometric data that can serve as valuable references for evaluating early morphometric features of graphs and axes.

In clinical practice, these growth curves can be included as standardized benchmarks in prenatal MRI protocols. Radiologists can quantitatively compare the measured ossification parameters of the atlas and axis with a specific reference range for gestational age. Deviation from the expected growth trajectory may be an early indicator of congenital abnormalities, such as atlantoaxial instability or underdevelopment. Incorporating these quantitative criteria into the MRI reporting framework can improve diagnostic accuracy and allow for systematic follow-up of high-risk pregnancies. Future developments, including AI-assisted segmentation, may further promote the automated application of these growth curves in routine prenatal imaging [11-15].

Although this study is based on autopsy specimens, which eliminates common motion artifacts in fetal MRI in vivo, we acknowledge that autopsy imaging is not directly equivalent to prenatal clinical MRI. Therefore, our results should be interpreted as baseline morphological measurement references under controlled conditions, rather than direct diagnostic criteria in clinical practice. In the future, research combining in vivo MRI is needed to validate and expand these findings.

Previous research on fetal spinal ossification mainly focused on general skeletal maturation or limited two-dimensional evaluation using radiography or ultrasound [16, 17]. Compared to these modes, MRI has significant advantages. Ultrasound is widely available and safe in prenatal imaging, but highly dependent on the operator and often limited by acoustic shadows, especially when evaluating deep or complex anatomical areas such as the cervical spine [18]. Although Computed Tomography (CT) provides excellent spatial resolution for bone structure, it exposes the fetus to ionizing radiation, making it unsuitable for routine prenatal assessment [19]. In contrast, MRI combines high soft tissue contrast, three-dimensional capability, and non-ionizing radiation, making it the preferred method for detailed assessment of fetal skeletal structure, including small ossification centers [8]. The ability of MRI to generate isotropic high-resolution datasets enables precise volume analysis, as demonstrated in this study, providing a non-invasive and safe tool for studying in situ fetal anatomy.

The strong linear correlation observed between gestational age and measurement parameters is consistent with the early findings of progressive fetal ossification, but it has been extended by providing specific growth equations for these key cervical structures [20, 21]. Importantly, the ossification of the lateral mass of the atlas, vertebral body, and axial arch follows different but parallel trajectories, indicating a coordinated but anatomically specific pattern of cervical maturity. A high coefficient of determination (R^2^values typically exceeding 0.70) supports the reliability of these growth trends. These morphometric benchmarks can assist in prenatal detection of abnormal ossification patterns, which may indicate congenital abnormalities such as atlantoaxial instability or cervical incompetence.

There is no significant gender difference in ossification dynamics, which is consistent with previous studies on fetal skeletal development, indicating that gender does not have a major impact on early vertebral ossification [22]. This discovery simplifies clinical interpretation, allowing for the application of a single growth criterion regardless of fetal gender.

Although this study has advantages, including the use of high-resolution imaging and strict measurement protocols with excellent interobserver consistency, some limitations must be acknowledged. Firstly, although the sample size of fetal imaging research is relatively large, it is still limited compared to population-based standards. Secondly, the cross-sectional nature of the study did not capture longitudinal changes within the same fetus. Thirdly, although CRL and LMP are used to confirm gestational age, the inherent small errors in these estimates cannot be completely ruled out.

In summary, this study provides comprehensive standardized data for the morphometric development of primary ossification centers of the fetal atlas and axis. Establishing a reliable growth model through non-invasive MRI imaging is expected to improve prenatal assessment of cervical spine development and early detection of congenital abnormalities.

## 5 LIMITATIONS

There are several limitations worth considering in this study. Firstly, although the sample size of 41 fetuses is relatively large for fetal imaging research, its universality at the population level is still limited. A larger-scale multicenter survey is needed to validate the findings of different populations. Secondly, although the estimation of gestational age based on Crown-Rump Length (CRL) and last menstrual period (LMP) is correlated, it may still introduce slight inaccuracies. Thirdly, although the reliability between observers and within observers is high, manual segmentation using 3D slicers essentially relies on operators, and future research can benefit from automated or AI-assisted analysis. Fourthly, the cross-sectional nature of the study did not capture longitudinal changes within the same fetus. We suggest conducting longitudinal MRI studies in the future to confirm ossification trajectories.

## CONCLUSION

This study provides standardized morphometric data for the main ossification centers of the atlas and axis in human fetuses, providing new insights into the normal growth trajectory of these key structures. These findings enhance our understanding of early cervical spine development, particularly limited to the atlas and axis. Although these results highlight the potential of MRI in the detailed assessment of fetal bones, they should be considered as baseline morphological measurement references derived from autopsy imaging. Future research needs to include additional vertebral levels and validate these findings using *in vivo* fetal MRI.

## Figures and Tables

**Fig. (1):**
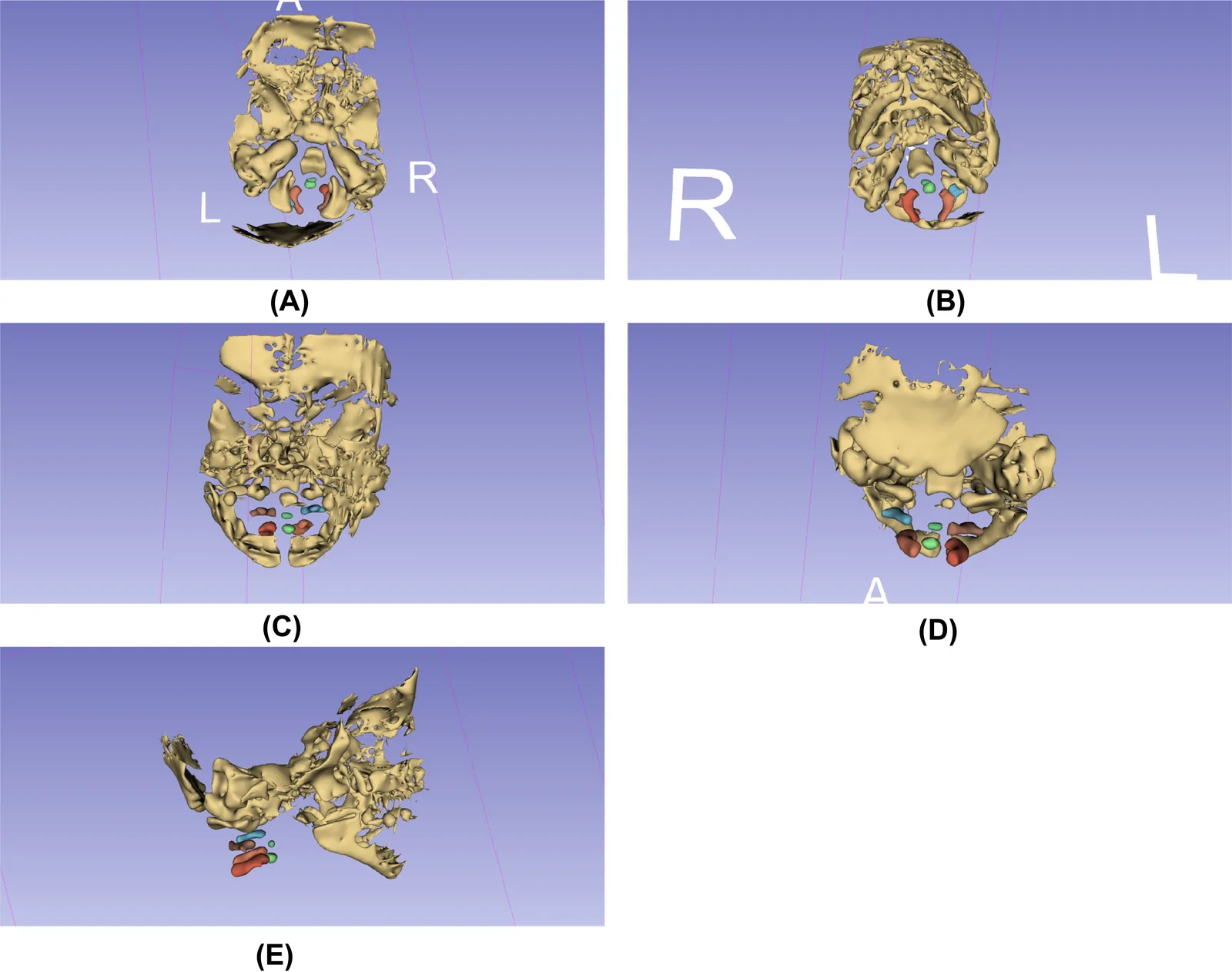
**A**)-three- dimensional reconstruction of ossification centres of the atlas and axis vertebrae in the MRI image of a female human foetus aged 25 weeks. A-view from above; **B**)-view from below; **C**)-view from the front; **D**)-view from the back; **E**)-view from the lateral side.

**Fig. (2):**
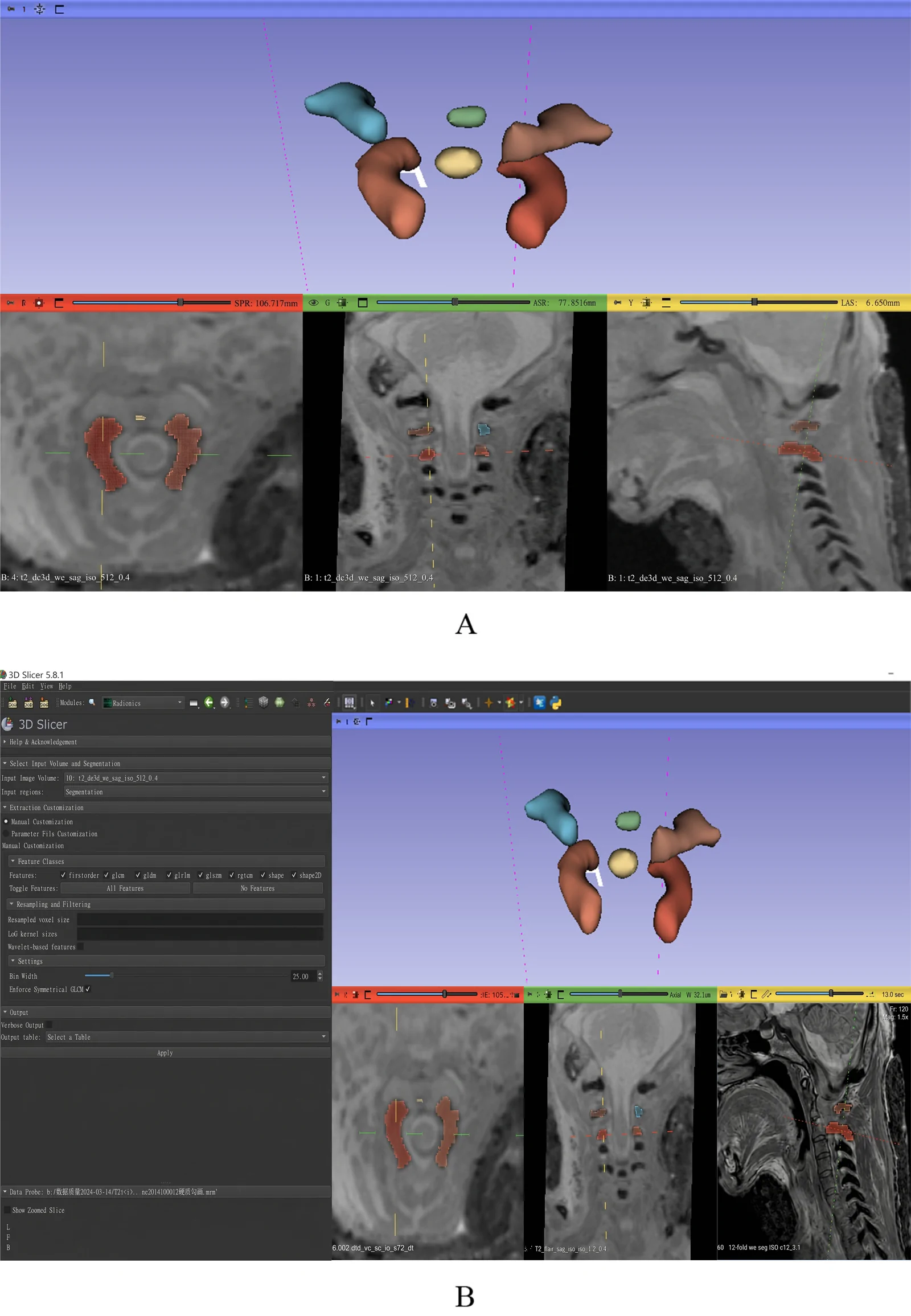
The illustration of the measurement methodology employed in this study. **A**)- the segment editor function of 3D-slicer 5.8.1 software was used to delineate the region of interest of the ossification center layer by layer and generate 3D images; **B**)-the data about the region of interest of the ossification center was exported through plug-in radiomics: 1. volume 2, projection surface area 3, maximum three-dimensional two-point distance. Due to the different placement positions of each fetal specimen and the inconsistency of the XYZ axes during MRI scanning, there were differences in the maximum distances exported in the coronal, sagittal, and transverse planes, which could not be included in the analysis. The maximum three-dimensional two-point distance was not affected by the position of the fetus during MRI scanning.

**Fig. (3):**
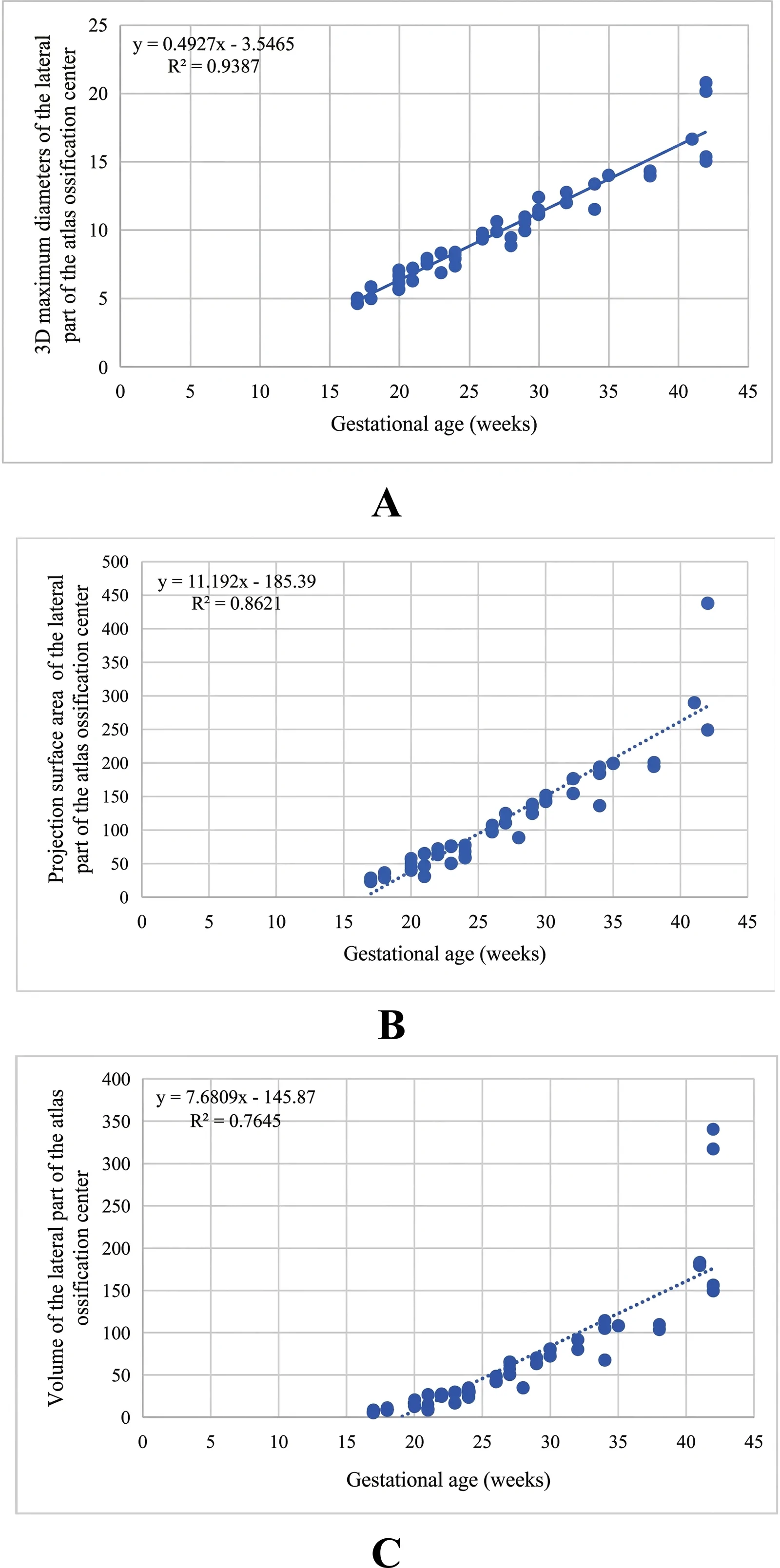
Regression lines for **A**)-3D maximum diameter, **B**)- projection surface area, **C**)-volume of ossification centres of the lateral parts of the atlas ossification center.

**Fig. (4):**
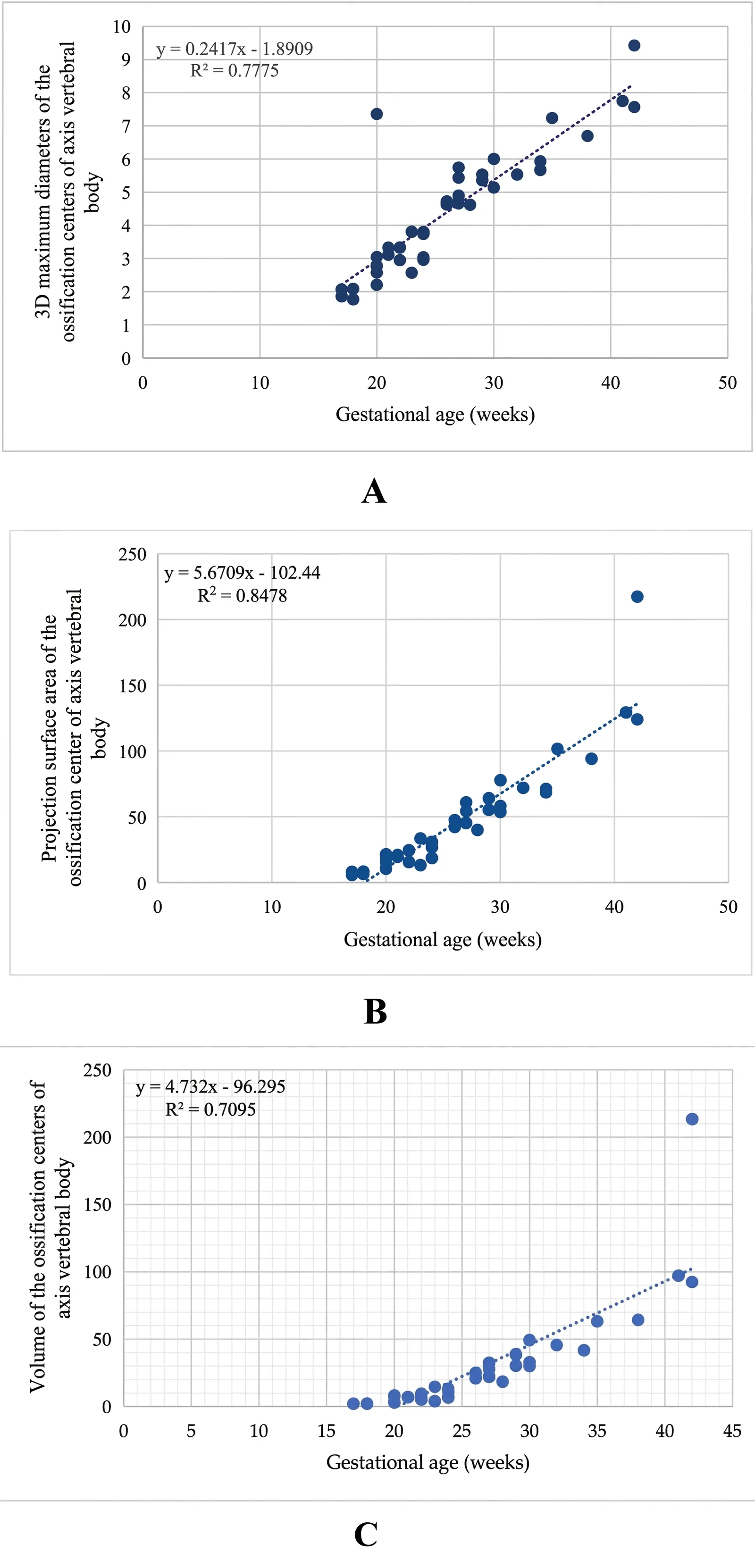
Regression lines for **A**)-3D maximum diameter, **B**)- projection surface area, **C**)-volume of ossification centres of the axis vertebral body.

**Fig. (5):**
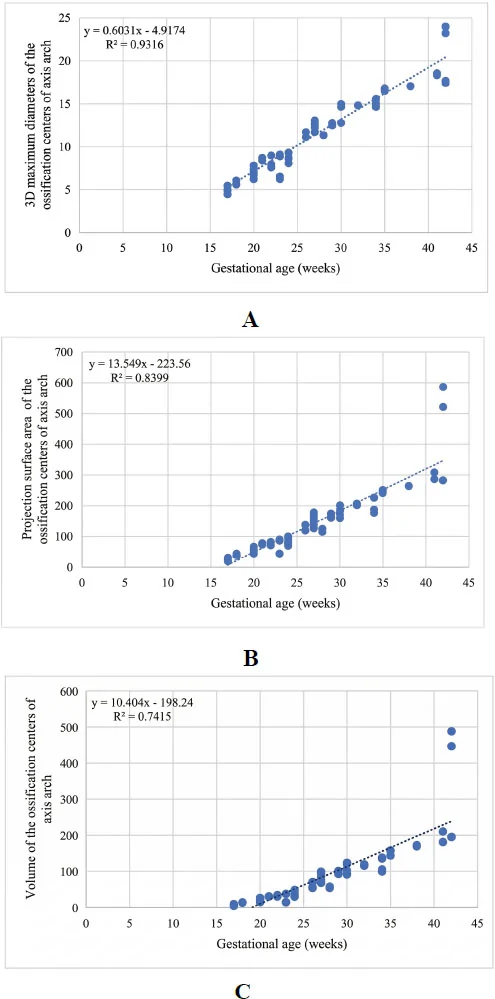
Regression lines for **A**)-3D maximum diameter, **B**)- projection surface area, **C**)-volume of ossification centres of the lateral parts of the axis arch.

**Table 1:** Age, number and sex of the foetuses studied.

**Gestational Age**	** Crown-rump Length of Foetuses (mm)**				**Number**	**Sex**	
	**Mean**	**SD**	**Minimum**	**Maximum**		**Male**	**Female**
17	128.55	2.77	126.78	130.31	2	2	0
18	138.35	1.76	135.65	138.35	2	1	1
20	160.96	3.87	156.43	164.78	6	4	2
21	172.77	2.62	170.06	174.94	2	1	1
22	183.57	4.45	178.87	188.27	2	1	1
23	196.38	3.78	192.56	200.20	2	2	0
24	206.18	2.98	203	209.36	4	2	2
26	225.79	3.67	222.76	228.82	2	1	1
27	238.60	5.72	232.87	244.33	4	1	3
28	247.40	-	247.40	247.40	1	1	0
29	255.21	1.23	253.45	256.97	2	1	1
30	264.01	0.78	262.56	265.34	3	2	1
32	287.62	-	287.62	287.62	1	1	0
34	307.23	0.49	306.67	308.24	3	2	1
35	323.04	-	323.04	323.04	1	1	0
38	355.45	-	355.45	355.45	1	0	1
41	386.87	-	386.87	386.87	1	0	1
42	398.67	-	398.67	398.67	2	1	1

**Table 2:** Intra-class correlation coefficients (ICC) values for inter-observer recurrence.

**Parameter**	**ICC**
3D maximum diameters of the lateral part of the atlas ossification center	0.946
3D maximum diameters of the ossification centers of axis arch	0.964
3D maximum diameters of the ossification centers of axis vertebral body	0.945
Projection surface area of the ossification centers of axis arch	0.928
Projection surface area of the ossification centers of axis vertebral body	0.929
Projection surface area of the lateral part of the atlas ossification center	0.958
Volume of the lateral part of the atlas ossification center	0.988
Volume of the ossification centers of axis arch	0.984
Volume of the ossification centers of axis vertebral body	0.993

**Table 3:** 3D maximum diameters of the lateral part of the atlas ossification center.

**Gestational Age**	**N**	** Lateral Part of the Ossification Centre**			
		**3D maximum diameter (mm)**			
		**Right**		**Left**	
		**Mean**	**SD**	**Mean**	**SD**
17	2	4.60	0.01	4.87	0.20
18	2	5.38	0.61	5.39	0.54
20	6	6.36	0.43	6.40	0.55
21	2	7.11	0.10	6.68	0.65
22	2	7.64	0.14	7.69	0.28
23	2	7.55	0.98	7.60	1.03
24	4	7.71	0.42	7.87	0.48
26	2	9.50	0.27	9.52	0.30
27	4	10.29	0.29	10.23	0.32
28	1	10.35		10.27	-
29	2	10.67	0.30	10.42	0.77
30	3	11.63	0.67	11.60	0.57
32	1	11.95	-	12.76	-
34	3	12.41	1.24	12.39	1.43
35	1	13.92	-	13.98	-
38	1	13.88	-	14.31	-
41	1	16.60	-	16.61	-
42	2	20.75	1.76	20.12	1.87

**Table 4:** Projection surface area and volume of the lateral part of the atlas ossification center.

**Gestational Age**	**N**	**Lateral Part of the Ossification Centre**							
		**Projection Surface Area (mm^2^)**				**Volume (mm^3^)**			
		**Right**		**Left**		**Right**		**Left**	
		**Mean**	**SD**	**Mean**	**SD**	**Mean**	**SD**	**Mean**	**SD**
17	2	23.24	1.85	26.37	3.82	6.37	1.04	7.50	2.13
18	2	33.24	5.18	32.39	5.66	9.95	2.21	9.91	2.42
20	6	46.78	5.34	45.88	6.70	15.99	2.53	15.76	3.45
21	2	56.20	14.07	46.65	22.51	21.76	8.27	17.54	12.82
22	2	65.25	4.14	67.72	6.86	25.54	0.00	26.69	2.51
23	2	63.13	15.75	63.07	20.45	23.62	7.36	24.39	10.01
24	4	68.00	8.84	68.71	8.30	27.62	4.60	28.43	5.07
26	2	99.90	4.38	104.41	3.68	43.49	2.08	46.37	3.50
27	4	119.28	6.15	116.99	6.18	58.87	5.42	56.31	6.73
28	1	123.45	-	125.87	-	64.41	-	65.83	-
29	2	130.23	6.17	131.05	10.59	65.84	3.47	67.32	5.59
30	3	146.73	5.14	147.78	5.86	77.18	4.90	78.06	1.76
32	1	154.32	-	176.59	-	80.42	-	91.65	-
34	3	165.82	39.70	179.20	34.59	91.85	32.48	96.45	27.00
35	1	198.45	-	199.03	-	109.50	-	107.55	-
38	1	201.08	-	204.18	-	110.95	-	113.63	-
41	1	288.52	-	290.19	-	183.03	-	179.65	-
42	2	340.04	45.76	333.84	53.78	240.66	42.76	216.98	39.43

**Table 5:** 3D maximum diameters, projection surface area and volume of the ossification centers of axis vertebral body.

**Gestational Age**	**N**	**The Ossification Centers of Axis Vertebral Body**					
		**3D Maximum Diameter (mm)**		**Projection Surface Area (mm^2^)**		**Volume (mm^3^)**	
		**Mean**	**SD**	**Mean**	**SD**	**Mean**	**SD**
17	2	1.97	0.14	7.16	1.30	1.46	0.57
18	2	1.93	0.23	7.58	1.09	1.66	0.35
20	6	3.42	1.94	15.76	4.09	4.79	2.02
21	2	3.23	0.16	20.25	1.10	6.84	0.21
22	2	3.15	0.27	20.18	6.39	7.08	3.16
23	2	3.19	0.86	23.39	14.02	9.19	7.60
24	4	3.39	0.44	23.74	6.22	9.14	3.43
26	2	4.68	0.08	44.81	3.96	22.86	3.10
27	4	5.18	0.49	51.42	7.57	26.39	5.25
28	1	5.22		54.13		28.12	
29	2	5.44	0.11	59.76	6.08	34.04	5.75
30	3	5.42	0.50	63.20	12.76	37.35	10.61
32	1	5.52		72.14		45.27	
34	3	5.80	0.18	79.65	1.96	51.78	0.06
35	1	7.22		101.73		63.17	
38	1	7.69		103.95		64.01	
41	1	7.74		128.90		96.95	-
42	2	8.41	0.78	157.21	10.75	113.31	8.76

**Table 6:** 3D maximum diameters of the ossification centers of axis arch.

**Gestational Age**	**N**	**The Ossification Centers of axis Arch**			
		**3D Maximum Diameter (mm)**			
		**Right**		**Left**	-
		**Mean**	**SD**	**Mean**	**SD**
17	2	4.84	0.63	5.16	0.43
18	2	5.82	0.21	5.77	0.32
20	6	7.12	0.39	7.01	0.55
21	2	7.54	0.21	7.66	0.02
22	2	8.22	0.95	8.47	0.74
23	2	8.71	1.61	8.62	2.06
24	4	8.81	0.61	8.79	0.44
26	2	11.37	0.46	11.39	0.38
27	4	12.32	0.59	12.55	0.37
28	1	12.28		12.29	-
29	2	12.53	0.08	12.68	0.08
30	3	14.16	1.23	14.07	1.11
32	1	14.76	-	14.73	-
34	3	14.98	0.60	15.19	0.48
35	1	16.49	-	16.79	-
38	1	17.04	-	16.98	-
41	1	18.53	-	18.32	-
42	2	20.79	4.43	20.27	4.06

**Table 7:** Projection surface area and volume of the ossification centers of axis arch.

**Gestational Age**	**N**	** The ossification centers of axis arch**							
		** Projection surface area (mm** **2** **)**				** Volume (mm** **3** **)**			
		**Right**		**Left**		**Right**		**Left**	
		**Mean**	**SD**	**Mean**	**SD**	**Mean**	**SD**	**Mean**	**SD**
17	2	22.57	6.39	25.00	7.41	5.32	2.54	6.17	3.43
18	2	38.70	3.75	40.13	5.04	11.93	1.26	12.83	2.08
20	6	53.61	7.36	53.76	8.44	19.35	3.83	19.32	4.59
21	2	75.54	3.36	75.57	1.50	28.52	1.11	28.89	2.05
22	2	76.07	8.29	75.83	6.41	31.53	3.49	31.45	3.22
23	2	73.72	29.89	77.69	31.56	35.08	16.26	33.85	16.94
24	4	85.33	15.48	82.38	11.52	37.73	9.74	36.06	6.39
26	2	125.59	10.81	128.55	13.45	61.51	11.88	63.45	9.57
27	4	142.27	18.77	154.72	17.71	78.43	12.31	82.19	13.75
28	1	155.40	-	162.85	-	82.98	-	88.66	-
29	2	164.31	7.21	169.11	6.57	95.08	4.97	96.99	5.85
30	3	178.67	19.21	183.39	16.76	102.89	15.24	106.64	16.31
32	1	206.73	-	199.91	-	119.69	-	114.71	-
34	3	220.03	34.20	205.71	25.91	128.69	28.17	120.05	19.66
35	1	240.26	-	250.24	-	144.16	-	155.11	-
38	1	263.40	-	262.09	-	173.43	-	168.24	-
41	1	308.46	-	284.56	-	210.50	-	181.69	-
42	2	435.14	62.37	400.66	49.87	341.80	50.54	320.24	32.67

## Data Availability

The authors confirm that the data supporting the findings of this research are available within the article.

## LIST OF ABBREVIATIONS

MRI: Magnetic resonance imaging
CRL: Crown–rump length
LMP: Last menstrual period
3D: Three-dimensional
TR: Repetition time
TE: Echo time
FOV: Field of view
DICOM: Digital imaging and communications in medicine
ANOVA: One-way analysis of variance
ICCs: Intra-class correlation coefficients.

## STANDARDS OF REPORTING

STROBE guidelines were followed.
